# Insights into the species-specific metabolic engineering of glucosinolates in radish (*Raphanus sativus* L.) based on comparative genomic analysis

**DOI:** 10.1038/s41598-017-16306-4

**Published:** 2017-11-22

**Authors:** Jinglei Wang, Yang Qiu, Xiaowu Wang, Zhen Yue, Xinhua Yang, Xiaohua Chen, Xiaohui Zhang, Di Shen, Haiping Wang, Jiangping Song, Hongju He, Xixiang Li

**Affiliations:** 1 Institute of Vegetables and Flowers, Chinese Academy of Agricultural Sciences; Key Laboratory of Biology and Genetic Improvement of Horticultural Crops, Ministry of Agriculture, Beijing, 100081 China; 2 Beijing Genomics Institute, Shenzhen, Guangdong, 518083 China; 3 Vegetable Research Center of the Beijing Academy of Agriculture and Forestry Sciences, Beijing, 100097 China

## Abstract

Glucosinolates (GSLs) and their hydrolysis products present in Brassicales play important roles in plants against herbivores and pathogens as well as in the protection of human health. To elucidate the molecular mechanisms underlying the formation of species-specific GSLs and their hydrolysed products in *Raphanus sativus* L., we performed a comparative genomics analysis between *R. sativus* and *Arabidopsis thaliana*. In total, 144 GSL metabolism genes were identified, and most of these GSL genes have expanded through whole-genome and tandem duplication in *R. sativus*. Crucially, the differential expression of *FMOGS-OX2* in the root and silique correlates with the differential distribution of major aliphatic GSL components in these organs. Moreover, *MYB118* expression specifically in the silique suggests that aliphatic GSL accumulation occurs predominantly in seeds. Furthermore, the absence of the expression of a putative non-functional *epithiospecifier* (*ESP)* gene in any tissue and the *nitrile-specifier* (*NSP*) gene in roots facilitates the accumulation of distinctive beneficial isothiocyanates in *R. sativus*. Elucidating the evolution of the GSL metabolic pathway in *R. sativus* is important for fully understanding GSL metabolic engineering and the precise genetic improvement of GSL components and their catabolites in *R. sativus* and other Brassicaceae crops.

## Introduction

Glucosinolates (GSLs), a large class of sulfur-rich secondary metabolites whose hydrolysis products display diverse bioactivities, function both in defence and as an attractant in plants, play a role in cancer prevention in humans and act as flavour compounds^1–4^. GSLs are present in the pungent plants of the order Brassicales, which consists of sixteen families^5^, including Brassicaceae, Capparidaceae, Caricaceae and Euphorbiaceae (specifically the genus *Drypetes*)^6^. There is great variation in GSL components and contents from species to species, with more than 200 types of GSLs identified^5,7^. These diverse GSLs have been classified into three major groups based on the structures of their various amino acid precursors: the aliphatic GSLs, derived from methionine, isoleucine, leucine or valine; the aromatic GSLs, derived from phenylalanine or tyrosine; and the indole GSLs, derived from tryptophan^2,8^. The basic biosynthesis pathway of aliphatic and aromatic GSLs comprises three phases: side-chain elongation, core structure formation and secondary modifications^9^.

The glucosinolate–myrosinase system involved in Brassicales secondary metabolism has been well studied. GSLs and myrosinase (thioglucoside glucohydrolase, EC 3.2.1.1471) are normally separated into the partitioned spaces of cells. GSLs can be enzymatically hydrolysed into several different types of breakdown products, such as isothiocyanates (ITCs), epithionitriles, and nitriles, which differ among species^10^, depending on the enzyme, substrate, pH, and presence of iron ions, while GSLs and myrosinase are encountered when tissues have been disrupted^11^. ITC, a pungent compound specific to Brassicaceae plants, is well known to exert antimicrobial activity against bacteria and fungi in plants^12,13^ and to effectively decrease the carcinogenic risk of colon and lung cancer^14,15^. In contrast, epithionitriles and nitriles have been shown to have little potential for conferring health-benefits^16,17^.

Radish (*Raphanus sativus* L., 2*n* = 2*x* = 18), a member of the Brassiceae tribe in the plant family Brassicaceae^14^, is a historically cultivated crop worldwide. *R. sativus* is a relative of *B. rapa* and *B. oleracea*. The main GSLs in the seeds of *B. rapa* and *B. oleracea* are progoitrin and gluconapin^18,19^, both of which are aliphatic; however, in the roots of *B. rapa* and *B. oleracea*, the predominant GSL is gluconasturtiin, which is aromatic^19^. Glucoraphenin, which accounts for 70–95% of the total GSLs in seeds of *R. sativus*, and glucoraphasatin, which can account for 50–90% of the total GSLs in roots, are aliphatic GSLs specific to *R. sativus* ^20–22^. Aliphatic GSLs are predominant in the seeds of *R. sativus*, which has also been demonstrated in *B. rapa* and *B. oleracea* ^18,19^. In *B. rapa* and *B. oleracea*, the major GSL hydrolysis products are nitriles and epithionitriles rather than ITCs^23,24^. In contrast, ITCs are preferentially produced in *R. sativus*, whereas a small amount of nitriles and epithionitriles are formed^25^.

In the model plant *A. thaliana*, many genes have been investigated in the context of controlling GSL biosynthesis^2,26^. Furthermore, in comparative studies with *A. thaliana*, the genes that control GSL biosynthesis in Brassicaceae vegetables have been identified and characterized. One hundred two and 105 GSL biosynthesis genes were identified in *B. rapa* ^27^ and *B. oleracea* ^28^, respectively. Moreover, 87 and 104 GSL biosynthesis genes were reported in two *R. sativus* genomes^29,30^, respectively. The greatest variation between the GSL profiles of *B. rapa, B. oleracea* and *R. sativus* is mainly attributed to the duplication or loss of two genes, *GRS1* and *AOP* ^28,29,31^. *GRS1* catalyses the dehydrogenation reaction to generate the unsaturated 4-carbon GSL from glucoerucin or glucoraphanin to obtain glucoraphasatin or glucoraphenin, respectively; these reactions are specific to *R. sativus* ^31^. *AOP2* and *AOP3* catalyse the formation of alkenyl and hydroxyalkyl GSLs, respectively^28^. Only one *AOP2* is functional in *B. oleracea*, and no *AOP2* homologue has been identified in *R. sativus*; however, three copies of *AOP2* have been identified in *B. rapa*, and no *AOP3* homologue has been identified in these three vegetables. Therefore, low amounts of sinigrin and gluconapin were found in *R. sativus* ^27–29^. Epithiospecifier protein (ESP) and nitrile-specifier protein (NSP) can catalyse GSL to produce nitriles and epithionitriles, respectively^32,33^, but the enzyme cofactor ESP is inactivated in *R. sativus* ^34^. Although several studies on *R. sativus* genes have involved GSL biosynthetic and degradation pathways^29–31^, the mechanism responsible for the existence and distribution of both species-specific GSLs and the hydrolysis products of these GSLs remains unclear, which leads to the questions of why *R. sativus* seeds show substantial accumulation of aliphatic GSLs rather than aromatic and indole GSLs and why low amounts of nitriles exist in *R. sativus*. Furthermore, the tissue-specific distribution of the two dominating GSLs (glucoraphenin and glucoraphasatin) remains to be elucidated in *R. sativus*.

In this study, we systematically identified GSL metabolism genes in *R. sativus*. Furthermore, we present several interesting observations that may explain the formation and distribution of species-specific GSLs and their hydrolysed products in *R. sativus*. The results of this study contribute to a more thorough understanding of how to precisely genetically modify and improvement of the the GSL metabolism pathway in *R. sativus* and its relatives.

## Results

### Identification and analysis of GSL genes in *R. sativus*

**Figure 1:**
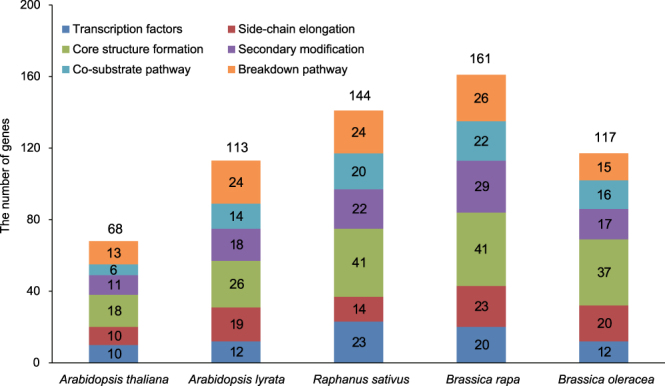
Numbers of GSL genes in *R. sativus* and related species. The numbers in the colour blocks represent the gene numbers in the corresponding sub-pathway categories. The numbers above the columns represent the total gene numbers in the corresponding species.

**Figure 2:**
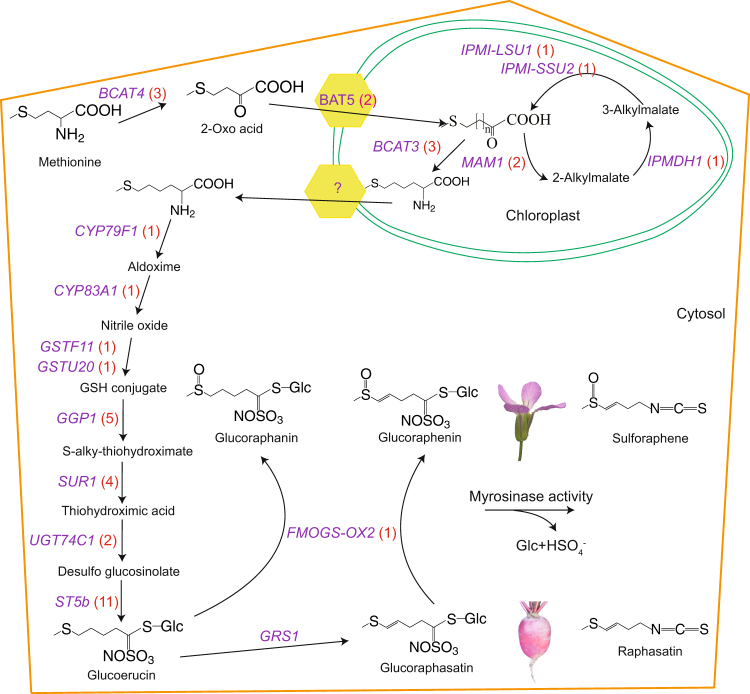
Putative aliphatic GSL biosynthetic and degradation pathways in *R. sativus*. Glucoraphasatin is principally found in the root of *R. sativus*, whereas glucoraphenin is detected mainly in the flowers. The genes shown in purple represent the genes involved in aliphatic GSL biosynthesis in *R. sativus*, and the numbers in the red brackets are the numbers of corresponding genes in *R. sativus*. The yellow polygon stands for the translocator on the chloroplast membrane. The symbol ‘?’ represents unknown genes.

By comparing the draft *R. sativus* genome^35^ with the *A. thaliana* genome, we discovered 144 GSL genes, which were slightly fewer than the 161 GSL genes present in *B. rapa*, greater than the 113 GSL genes of *Arabidopsis lyrata* and the 117 GSL genes of *B. oleracea*, and two-fold higher than the 68 found in *A. thaliana* (Fig. 1 and Supplementary Table S1). Notably, *R. sativus* homologues corresponding to 17*A. thaliana* GSL genes were not identified in this study. These *A. thaliana* genes include *MAM3*, *IPMI-SSU3*, *IPMDH3, CYP79F2*, three *FMOGS-OX* genes and two transcription factors (Supplementary Table S1). In addition, *TGG2* was not found in the ‘XYB36-2’ genome, but two *TGG2* genes were reported by Jeong *et al*.^30^. Regarding gene numbers, there was less variation, with 40% more side-chain elongation genes observed in *R. sativus* compared to *A. thaliana*, whereas in the co-substrate pathway, the number of genes found in *R. sativus* was 2.33 times the number present in *A. thaliana* (Fig. 1). As aliphatic GSLs are the major GSLs present in *R. sativus*, the loss, retention and expansion of the GSL genes may have led to the accumulation of species-specific GSLs in *R. sativus* (Fig. 2).

### Expansion of the GSL metabolic genes in *R. sativus*

Gene copy numbers can be expanded in four major ways: genome duplication, segmental duplication, tandem duplication and via transposable elements^36^. Most of the retained genes (36 out of 51) were present as multi-copies in *R. sativus* (Supplementary Table S1), which may have resulted from tandem duplication or whole-genome triplication (WGT) occurring after the divergence of *R. sativus* and *A. thaliana*. Of the 36 expanded genes, 25 AtGSL genes have syntenic relationships to 51 RsGSL genes (Supplementary Table S2). Furthermore, 16 of the 36 over-retained AtGSL genes were tandemly duplicated and distributed in 17 tandem arrays in *R. sativus* (Supplementary Table S3). These data showed that tandem duplication and WGT substantially contributed to the expansion of GSL metabolic genes in *R. sativus*.

We analysed the number and the ratio of single-copy to multi-copy homologous genes to reveal the retention status of the three types of GSL core structure formation genes in *R. sativus* after the WGT. The gene copy number ratios between *R. sativus* and *A. thaliana* were 2 (6:3), 0.8 (5:4) and 1 (4:4) for aliphatic, indole and aromatic GSL core structure formation genes, respectively (Table 1). Furthermore, the downstream genes (*GGP1*, *SUR1*, *UGT74C1* and *ST5b*), which retained more than one copy, were more frequently duplicated than upstream genes in the pathway for aliphatic GSL core structure biosynthesis in *R. sativus* (Fig. 2).

**Table 1:** Number and ratio of single-copy to multi-copy homologues of GSL core structure formation genes in *R. sativus*.

Types of GSLs	Number of homologues with different copies					Ratio of single to multiple copies
	0	1	2	3	Total	
aliphatic GSLs	1	3	1	5	10	3:6
indole GSLs	0	5	2	2	9	5:4
aromatic GSLs	0	4	2	2	8	4:4

**Figure 3:**
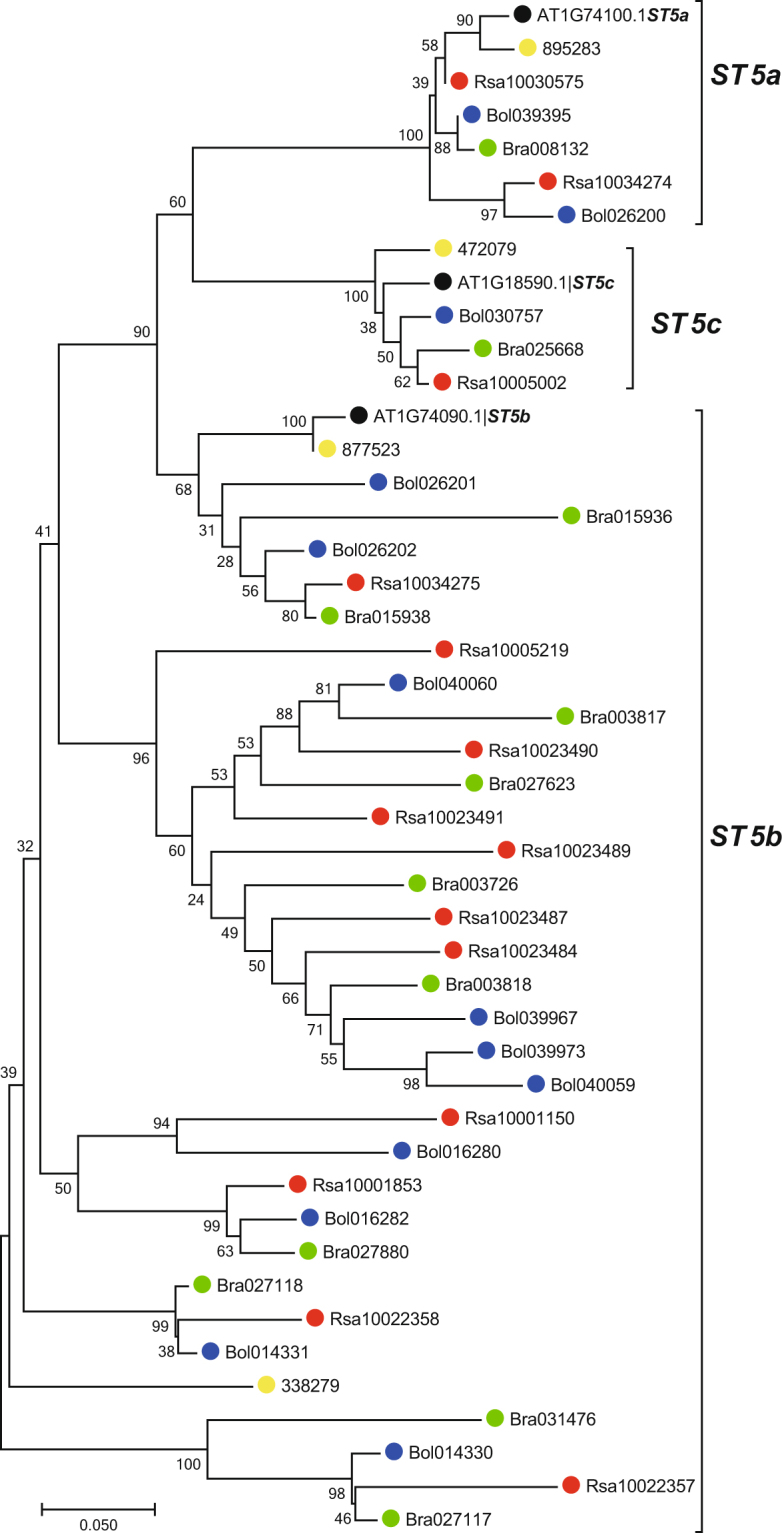
Phylogenetic tree of the *ST* genes in *R. sativus* and four related species. The full-length amino acid sequences were aligned with ClustalW, and the NJ tree was constructed with MEGA using 1000 bootstrap replicates. Each *ST* gene is indicated along the lines on the right. *R. sativus* proteins are marked with solid red dots. The solid black, yellow, green and blue dots represent *A. thaliana, A. lyrata*, *B. rapa* and *B. oleracea*, respectively.

Notably, the *ST* genes were found to be highly expanded in *R. sativus*, and most of the *ST* copies (8 out of 14) in *R. sativus* were tandemly duplicated. *R. sativus* contained 11 copies of the *ST5b* gene, which encodes desulfoglucosinolate sulfotransferase, which is involved in the final step of GSL core structure biosynthesis^37^. The phylogenetic tree of the *ST* genes in *R. sativus* and its closely related species revealed that *ST5a* and *ST5b*, especially the latter, have undergone the greatest amount of expansion in *R. sativus*, *B. rapa* and *B. oleracea*. The copies of *ST5b* were clustered into three groups, two of which included only *ST5b* copies from *R. sativus*, *B. rapa* and *B. oleracea*, implying that they were inherited from a common ancestor and diverged via tandem duplication (Fig. 3).

### Expression patterns of *FMOGS-OX* and *MYB118* genes in *R. sativus*

**Figure 4:**
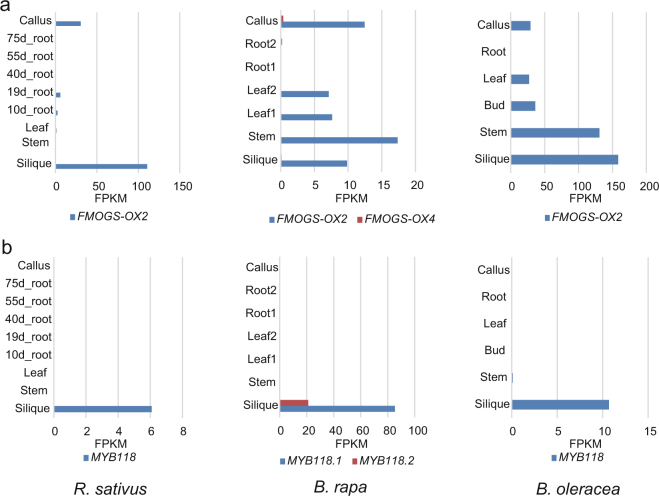
Expression levels of *FMOGS-OX2* and *MYB118*. (**a**) Expression levels of *FMOGS-OX* genes in various tissues of *R. sativus*, *B. rapa* and *B. oleracea*. (**b**) Expression levels of *MYB118* in various tissues of *R. sativus*, *B. rapa* and *B. oleracea*. The coloured scale bar represents the FPKM values.

*FMOGS-OX1-4* S-oxygenates both short- and long-chain methylthioalkyl GSLs to produce the corresponding methylsulfinylalkyl GSL^38^. Only *FMOGS-OX2* was identified as a single copy gene in *R. sativus*. Transcriptome analysis indicated that *FMOGS-OX2* is highly expressed in the silique but minimally expressed in the other tissues of *R. sativus*. *FMOGS-OX2* was also retained as one copy in *B. rapa* and *B. oleracea* and is expressed in all tissues except the roots. In addition, one *FMOGS-OX4* gene was identified in *B. rapa*, but this gene was minimally expressed in all tissues (Fig. 4a).

*MYB115* and *MYB118* play key roles in the aliphatic GSL biosynthetic pathway, acting as negative regulators in the benzoyloxy GSL pathway^39^. We have found that *MYB115* is lost but that *MYB118* is present in 1, 2 and 1 copies in *R. sativus, B. rapa* and *B. oleracea*, respectively. Interestingly, *MYB118* was expressed only in the silique in *B. rapa* and *R. sativus* and was expressed highly in the silique but at low levels in other tissues in *B. oleracea* (Fig. 4b).

### Putative non-functional *ESP* and tissue-specific expression of *NSP* genes in *R. sativus*

**Figure 5:**
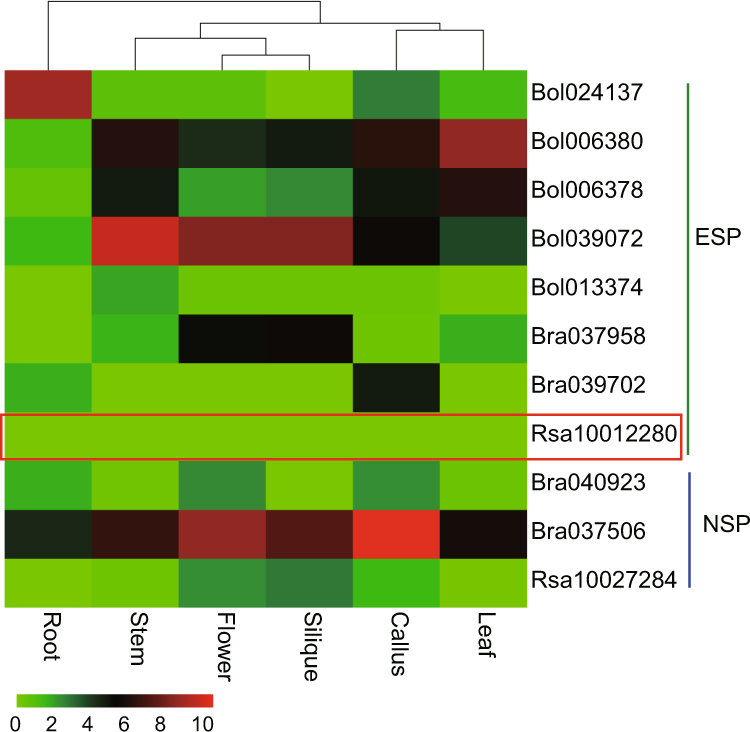
Expression of the *ESP* and *NSP* genes in *R. sativus*, *B. oleracea*, *B. rapa* and *A. thaliana*. Expression analysis of the *ESP* and *NSP* genes in: root, stem, flower, silique, callus and leaf tissues. The coloured scale bar in the upper right corner of the figure represents the log2 of FPKM values.

When tissues of Brassicales plants are damaged, myrosinase is released, and glucosinolates are degraded to isothiocyanate, thiocyanate, or nitrile derivatives. Although two *R. sativus* myrosinase genes have been cloned by Hara *et al*.^40^ and a total of 11*R. sativus* myrosinase genes were identified by Mitsui *et al*.^29^, it remains unclear why non-nitrile products exist in *R. sativus*. We found one *ESP* gene in *R. sativus*; we also identified two copies of *ESP* in *B. rapa* and five copies in *B. oleracea*. The RNA-seq data indicated that the *ESP* genes were not expressed in *R. sativus* but were expressed in both *B. rapa and B. oleracea* (Fig. 5). *NSP*, whose product promotes the breakdown of glucosinolates into nitrile derivatives, was found to be retained as one copy in *R. sativus* and as two copies in *B. rapa*. Interestingly, in *R. sativus*, the *NSP5* gene was expressed at a minimal level in the flowers, seedpods and callus and was not expressed in the roots, but one of the copies in *B. rapa* was highly expressed (Fig. 5).

## Discussion

We identified the counterparts of most of the *A. thaliana* GSL metabolic genes, which are present in various copy numbers in *R. sativus*. Compared with the GSL genes identified in two previously reported genomes, the copy numbers were the same for most of the identified genes^29,30^, with the exception of *TGG2*. The two *R. sativus TGG2* genes reported by Jeong *et al*.^30^ are not considered to be homologues, since these two genes were identified by using homologue searches based on the sequence of AT4G11150, which does not encode the *TGG2* gene in *A. thaliana*. The corresponding homologue of *TGG2* (Rs303830) identified by Jeong *et al*.^30^ were identified in the ‘XYB36-2’ and ‘Aokubi’ genomes, but they represented the homologues of *TGG1* in these two genomes^29^.

The qualitative and quantitative changes in the GSL profiles of *R. sativus* may be due to the loss, expansion, non/neo-functionality and tissue/stage-specific expression of GSL genes. According to our comprehensive and comparative analysis based on genome and transcriptome data from this study and previous research, these changes corresponded to the evolution of the *R. sativus* genome^29–31,35^. In terms of side-chain elongation, the absence of *MAM3* led to a lack of long-chain aliphatic GSLs in *R. sativus*, and this result is consistent with Mitsui *et al*.^29^. We also found that the loss of *CYP79F2*, of which the knockout decreases the abundance of long-chain aliphatic GSLs in *A. thaliana*, may also result in short-side-chain aliphatic GSLs in *R. sativus* ^41,42^. Considering that functional *MAM3* genes were found in *B. rapa* and *B. oleracea*, the loss of *CYP79F2* might be the main reason for the predominance of short-side-chain aliphatic GSLs observed in these two species. In addition, no homologues of *IPMI-SSU3* or *IPMDH3* from *A. thaliana* were found in *R. sativus*. The IPMIs, which are composed of a large subunit and a small subunit, catalyse the isomerization reaction. *IPMI-SSU3* encodes the small subunit. However, knockout mutants of *IPMI-SSU3* result in small changes in GSL levels in *A. thaliana* ^43^. IPMDH3 is a predicted enzyme that has a similar function to IPMDH1^44^, which promotes glucosinolate accumulation^45,46^. Therefore, the loss of *IPMI-SSU3* and *IPMDH3* is likely to be offset by *IPMI-LSU1, IPMI-LSU2* and *IPMDH1*.

Moreover, duplicated genes may enhance the potential for the quantitative variation of a particular trait^47^. Interestingly, most of the GSL genes were present in multiple copies. Syntenic analysis demonstrated that GSL genes have increased their copy numbers through WGT. In addition to WGT, some genes also expanded in number through tandem duplication, such as *ST5b*, which is present in 11 copies in *R. sativus*. Notably, expansion was observed for more aliphatic GSL core structure formation genes than for indole and aromatic GSL genes. Further, the biosynthesis genes for the core structures of aliphatic GSLs that were present in downstream locations were more over-retained than those in upstream locations. The redundancy of the downstream genes may guarantee products for successful synthesis of aliphatic GSLs.

Furthermore, with respect to the secondary modification step, three *FMOGS-OX* genes (*FMOGS-OX1*, *FMOGS-OX3* and *FMOGS-OX4*) and two *AOP* genes (*AOP2* and *AOP3*) were lost. *FMOGS-OX1-4* S-oxygenates both short- and long-chained methylthioalkyl GSLs to produce the corresponding methylsulfinylalkyl GSLs^38^. In *R. sativus*, we identified one copy of *FMOGS-OX2*, which catalyses the S-oxygenation of glucoraphasatin and converts glucoraphasatin into glucoraphenin. The results of the GSL content analyses performed in previous studies^21,22,48–50^ demonstrated that glucoraphasatin is predominant in the roots and leaves of *R. sativus*, whereas glucoraphenin accumulates heavily in the seeds. Correspondingly, *FMOGS-OX2* was highly expressed in the silique and minimally expressed in roots. The tissue-specific differential expression of *FMOGS-OX2* clearly results in the different predominant GSLs in various tissues of *R. sativus*. The *FMOGS-OX2* gene of *R. sativus* shows biotechnological potential, as the cancer-preventive properties of the plant involve sulforaphene, which is the catabolite of glucoraphenin^51^. Additionally, we found no *AOP2* or *AOP3* genes in *R. sativus*, as reported by Mitsui *et al*.^29^. The loss of these two genes prevents the conversion of S-oxygenated and hydroxyalkyl GSLs into downstream GSLs, which is the reason for the accumulation of glucoraphasatin and glucoraphenin in *R. sativus*.

In addition, two transcription factors (*MYB76*, *MYB115*) were lost in *R. sativus*. Although aliphatic GSL levels will increase with increased *MYB76* expression levels, a knockout mutant of *MYB76* was reported to exhibit no significant change in GSLs in *A. thaliana* ^52^. *MYB115* and *MYB118* are functionally redundant and interact to control the expression of GLS genes^39^. Therefore, the loss of *MYB76* and *MYB115* does not completely abolish GSL biosynthesis. While *MYB115* and *MYB118* negatively regulate aliphatic GSLs in *A. thaliana* ^39^, the double *myb115-myb118* mutant exhibits increased levels of most of the short-chain aliphatic GSLs with the exception of 4-methylthiobutyl GSL (glucoerucin, GER)^39^. Moreover, GER content increased in the single *myb115* mutant but significantly decreased in the *myb118* mutant^39^. That is, *MYB118* promotes the accumulation of GER but decreases the levels of other aliphatic GSLs in *A. thaliana*. GER is a precursor of the predominant aliphatic GSLs in *R. sativus*, *B. rapa* and *B. oleracea* ^53^. Several studies have shown that aliphatic GSLs, especially GER and its downstream products, are highly predominant in seeds in the three *Brassica* vegetables. In addition, in other organs, the percentages of aliphatic GSLs are decreased^18,19,53^. Considering that *MYB118* was expressed only in the silique, we inferred that its silique-specific expression results in the high accumulation of aliphatic GSLs in seeds of these three vegetables. Nonetheless, further work is required to elucidate the molecular basis of the regulation of GSL biosynthesis through *MYB118*.

The ESP and NSP proteins divert the myrosinase-catalysed hydrolysis of an aliphatic GSL from the formation of ITC to the production of epithionitrile and nitrile^54^. O’Hare *et al*. found that there were no ESP proteins in *R. sativus* ^34^. We identified one *ESP* gene showing no detectable expression in any organ of *R. sativus*, indicating that its function might have been lost in *R. sativus*. We also observed one *NSP5* gene with no expression profile in the roots and minimal expression in other organs, and we found no *NSP1-4* genes within the *R. sativus* genome. The non-expression of the *ESP* gene and the lack of expression of the *NSP* gene in the roots and the leaves were inferred to be the major reasons for the accumulation of distinctive beneficial isothiocyanates, such as sulforaphene, rather than epithionitriles or nitriles.

## Materials and Methods

### Data resources

The *A. thaliana* genome and annotation data were downloaded from TAIR10 (http://www.arabidopsis.org/)^55^. *B. rapa* and *B. oleracea* assembly and annotation data were downloaded from the BRAD database (http://brassicadb.org/brad/)^56^. The genome data for *A. lyrata* were obtained from the JGI database. The three whole-genome sequences of *R. sativus* sequenced by Zhang *et al*.^35^, Mitsui *et al*.^29^ and Jeong *et al*.^30^ were downloaded from the BRAD database^56^, NODAI Radish genome database (http://www.nodai-genome-d.org/)^29^ and Radish Genome database (http://www.radish-genome.org/)^30^, respectively. *B. rapa* and *B. oleracea* RNA-seq data were obtained from the Gene Expression Omnibus (GEO) database with accession numbers GSE43245 and GSE42891, respectively. *R. sativus* RNA-seq data^35^ are available at EMBL/NCBI/SRA (PRJNA413464).

### GSL gene identification in *R. sativus*

The GSL genes of *A. thaliana* have been reported previously^9,27,57,58^ and were aligned with corresponding protein sets from *R. sativus* ^35^, *B. rapa* ^59^, *B. oleracea* ^28^ and *A. lyrata* ^60^ using BLASTP (*E*-value ≤ 1 × 10^−10^, identity ≥55).

### Analysis of GSL genes in *R. sativus*

To detect the generation mechanism of the expanded genes, we identified syntenic orthologues using SynOrths based on both sequence similarity and the collinearity of flanking genes^61^. We identified tandem duplicate genes using the same standard with GSL gene identification, and only one unrelated gene was allowed to exist between the two genes in a tandem array^62^. CLUSTALW^63^ was employed for sequence alignment. The phylogenetic tree of the *ST* gene family members of *R. sativus* and other species was constructed using the neighbour-joining method with MEGA (version 7.0.21) software^64^.

## Electronic supplementary material

Supplementary Information
