# Supplementary material for: Insights into the species-specific metabolic engineering of glucosinolates in radish (Raphanus sativus L.) based on comparative genomic analysis
Source: Sci Rep. 2017 Nov 22;7:16040. doi: 10.1038/s41598-017-16306-4 (PMC5700054; doi:10.1038/s41598-017-16306-4)
Supplement: Supplementary file 1 — Supplementary Information [file 41598_2017_16306_MOESM1_ESM.doc]

**Title:** **Insights into the species-specific metabolic engineering of glucosinolates in radish (*Raphanus sativus* L.) based on comparative genomic analysis**

**Authors:** Jinglei Wang[[1]](#footnote-2)*, Yang Qiu^1*^, Xiaowu Wang^1^, Zhen Yue^2^, Xinhua Yang^2^, Xiaohua Chen^1^, Xiaohui Zhang^1^, Di Shen^1^, Haiping Wang^1^, Jiangping Song^1^, Hongju He^3^ & Xixiang Li^1^

**Supplementary Information**

**Table S1.** GSL genes reported in *Arabidopsis thaliana* and homologues identified in *Arabidopsis lyrata, Raphanus sativus, Brassica rapa* and *Brassica oleracea*.

|  | Genes | *Arabidopsis thaliana* | *Arabidopsis lyrata* | *Raphanus sativus* | *Brassica rapa* | *Brassica oleracea* |
| --- | --- | --- | --- | --- | --- | --- |
| Transcription factors | OBP2 | AT1G07640.3 | 878939;481796 | Rsa10041327;Rsa10003296;Rsa10019875 | Bra030696;Bra031588;Bra035667 | Bol023400;Bol041144;Bol006511 |
|  | IQD1 | AT3G09710.2 | 872328;487077 | Rsa10034552;Rsa10042918;Rsa10008778;Rsa10007931 | Bra034081;Bra001299 | Bol033935;Bol000775;Bol010076 |
|  | MYB28 | AT5G61420.2 | 919385 | Rsa10017936;Rsa10019636;Rsa10033628 | Bra035929;Bra012961;Bra029311 |  |
|  | MYB29 | AT5G07690.1 | 487604 | Rsa10020837;Rsa10036406 | Bra005949 |  |
|  | MYB34 | AT5G60890.1 | 496240 | Rsa10018401;Rsa10033651;Rsa10019673;Rsa10019672 | Bra013000;Bra035954;Bra029350;Bra029349 | Bol017062;Bol007760 |
|  | MYB51 | AT1G18570.1 | 472077 | Rsa10013845;Rsa10040860;Rsa10038229 | Bra016553;Bra025666;Bra031035 | Bol013207;Bol030761 |
|  | MYB76 | AT5G07700.1 | 861189 |  |  |  |
|  | MYB115 | AT5G40360.1 | 330471 |  |  |  |
|  | MYB118 | AT3G27785.1 | 484568 | Rsa10027175 | Bra033027;Bra025299 | Bol031189 |
|  | MYB122 | AT1G74080.1 | 476550 | Rsa10030571;Rsa10034277;Rsa10030572 | Bra015939;Bra008131 | Bol026204 |
| Side-chain elongation | BCAT3 | AT3G49680.1 | 485328;919922 | Rsa10032850;Rsa10006817;Rsa10037623 | Bra017964;Bra029966 | Bol037342;Bol021961 |
|  | BCAT4 | AT3G19710.1 | 898360;474144;474143;891934 | Rsa10031835;Rsa10031513;Rsa10006668 | Bra022448;Bra001761;Bra018831;Bra014235 | Bol018130;Bol026690;Bol015595;Bol045055;Bol011774 |
|  | BAT5 | AT4G12030.2 | 490020;914321 | Rsa10009388;Rsa10025621 | Bra000760;Bra029434;Bra019352 |  |
|  | IPMI-LSU1 | AT4G13430.1 | 883531 | Rsa10016403 | Bra040341;Bra032708 | Bol033454;Bol040343 |
|  | IPMI-SSU2 | AT2G43100.1 | 483411 | Rsa10039255 | Bra004744 | Bol029979 |
|  | IPMI-SSU3 | AT3G58990.1 | 907390;483410 |  | Bra004743 | Bol029441 |
|  | IPMDH1 | AT5G14200.1 | 488253;477193;895936 | Rsa10025187;Rsa10003435 | Bra023450;Bra035169;Bra008464;Bra008466 | Bol004236;Bol000668;Bol040564;Bol040562 |
|  | IPMDH3 | AT1G31180.1 | 473708 |  | Bra008463;Bra035169 | Bol040561 |
|  | MAM1 | At5g23010.1 | 910306 | Rsa10019680;Rsa10018392 | Bra018524;Bra029355 | Bol017070;Bol020647;Bol037823;Bol017071 |
|  | MAM3 | At5g23020.1 | 910307;326599 |  | Bra021947;Bra013011 |  |
| Core structure formation | CYP79A2 | AT5G05260.1 | 325159;856142 | Rsa10002991;Rsa10020966 | Bra028764;Bra009100 | Bol044048 |
|  | CYP79B2 | AT4G39950.1 | 481097 | Rsa10027010;Rsa10009991 | Bra030246 | Bol031784 |
|  | CYP79B3 | AT2G22330.1 | 490780 | Rsa10037345;Rsa10026308 | Bra017871;Bra011821;Bra010644 | Bol018585;Bol028852;Bol032767 |
|  | CYP79F1 | AT1G16410.1 |  | Rsa10038067 | Bra026058 | Bol038222 |
|  | CYP79F2 | AT1G16400.1 | 312617;889032 |  |  |  |
|  | CYP83A1 | AT4G13770.1 | 864718;913321 | Rsa10030406 | Bra016908;Bra032734 | Bol040365 |
|  | CYP83B1 | AT4G31500.1 | 493537 | Rsa10004699 | Bra034941 | Bol033477 |
|  | GSTF9 | AT2G30860.1 | 482003 | Rsa10032310 | Bra022815;Bra021673 | Bol033376;Bol004624 |
|  | GSTF10 | AT2G30870.1 | 482004 | Rsa10050001 | Bra022816 | Bol004625 |
|  | GSTF11 | AT3G03190.1 | 477565;488585 | Rsa10003824;Rsa10015104;Rsa10018713 | Bra032010;Bra023602;Bra008570 | Bol000843;Bol021325;Bol019821 |
|  | GSTU20 | AT1G78370.1 |  | Rsa10029860 | Bra003645 | Bol021558 |
|  | GGP1 | AT4G30530.1 | 491698;868204;328661;353756 | Rsa10010315;Rsa10019051;Rsa10023138;Rsa10023137;Rsa10010316 | Bra010283;Bra011201;Bra024068;Bra039220;Bra010282 | Bol012989;Bol018073;Bol033672;Bol044705 |
|  | SUR1 | AT2G20610.1 | 480895;491928;481345 | Rsa10031178;Rsa10022084;Rsa10022083;Rsa10036844 | Bra036490;Bra011026;Bra036703;Bra024204 | Bol038767;Bol033528 |
|  | UGT74B1 | AT1G24100.1 | 889955 | Rsa10040057 | Bra024634 | Bol005786 |
|  | UGT74C1 | AT2G31790.1 | 902214 | Rsa10016624;Rsa10038489 | Bra021743;Bra005641 | Bol014127;Bol006450 |
|  | ST5a | AT1G74100.1 | 895283 | Rsa10030575;Rsa10034274 | Bra008132;Bra003726 | Bol039395;Bol026200 |
|  | ST5b | AT1G74090.1 | 877523;338279 | Rsa10034275;Rsa10001853;Rsa10023491;Rsa10023490;Rsa10023484;Rsa10005219;Rsa10023487;Rsa10022358;Rsa10001150;Rsa10023489;Rsa10022357 | Bra015938;Bra003818;Bra015936;Bra027880;Bra027623;Bra027118;Bra027117;Bra003817;Bra031476 | Bol026202;Bol026201;Bol016282;Bol040060;Bol016280;Bol014331;Bol014330;Bol039967;Bol040059;Bol039973 |
|  | ST5c | AT1G18590.1 | 472079 | Rsa10050002 | Bra025668 | Bol030757 |
| Secondary modification | FMOGS-OX1 | AT1G65860.1 |  |  |  |  |
|  | FMOGS-OX2 | AT1G62540.1 | 315143 | Rsa10033546 | Bra027035 | Bol010993 |
|  | FMOGS-OX3 | AT1G62560.1 | 475092 |  |  |  |
|  | FMOGS-OX4 | AT1G62570.1 | 893225 |  | Bra027032 |  |
|  | FMOGS-OX5 | AT1G12140.1 | 334507;879464;888541;475086;471366;475088;475091 | Rsa10041588;Rsa10041089;Rsa10011674 | Bra026988;Bra016787;Bra019747;Bra026986 | Bol029100;Bol031350;Bol010425;Bol031354;Bol010992 |
|  | AOP1 | AT4G03070.1 |  | Rsa10009537;Rsa10022481;Rsa10022483;Rsa10031755 | Bra034182;Bra034181;Bra000847;Bra018992 | Bol030627;Bol030626 |
|  | AOP2 | AT4G03060.1 |  |  | Bra034180;Bra018521 |  |
|  | AOP3 | AT4G03050.2 |  |  |  |  |
|  | GSL-OH | AT2G25450.1 | 482001;470684;878835;878834 | Rsa10016552;Rsa10037760;Rsa10038425;Rsa10019929;Rsa10016553;Rsa10017523 | Bra022920;Bra021670;Bra021671;Bra021672;Bra031533;Bra031532;Bra015513;Bra015515;Bra007607 | Bol004155 |
|  | CYP81F2 | AT5G57220.1 | 332020;490876 | Rsa10023813;Rsa10019374;Rsa10019377;Rsa10026366;Rsa10026367 | Bra020459;Bra006830;Bra011761;Bra011762 | Bol026044;Bol014239;Bol028914;Bol028913 |
|  | CYP81F4 | AT4G37410.1 | 490879;328029 | Rsa10026964;Rsa10026966;Rsa10026369 | Bra010598;Bra011759;Bra010597;Bra011758 | Bol032712;Bol028918;Bol032711;Bol028919 |
| Co-substrate pathway | BZO1 | AT1G65880.1 | 475714 | Rsa10009399;Rsa10015752;Rsa10033891 | Bra038189;Bra004132;Bra039743 | Bol003620;Bol012559;Bol045054 |
|  | APK1 | AT2G14750.1 | 480365 | Rsa10041726 | Bra039818;Bra013120;Bra024443 | Bol019336;Bol042602 |
|  | APK2 | AT4G39940.1 | 912483;496843 | Rsa10027011;Rsa10037346;Rsa10026307 | Bra011822;Bra017872;Bra010645 | Bol018584;Bol028851;Bol043870;Bol032769 |
|  | CHY1 | AT5G65940.1 | 920141;481983;869334 | Rsa10016044;Rsa10010245;Rsa10007228;Rsa10020308;Rsa10020303 | Bra031802;Bra039968;Bra018392;Bra039975;Bra018395 | Bol000634;Bol017594;Bol033283;Bol033277 |
|  | GSH1_PAD2 | AT4G23100.1 | 492552;914292;864788 | Rsa10014287;Rsa10036547;Rsa10036546 | Bra013675;Bra019332;Bra019333 | Bol014965;Bol042077;Bol042075 |
|  | AAO4 | AT1G04580.1 | 470440;481603;323092;489007 | Rsa10020170;Rsa10023010;Rsa10021081;Rsa10013190;Rsa10028431 | Bra015330;Bra019448;Bra020140;Bra002347;Bra034975 |  |
| Breakdown pathway | TGG1 | AT5G26000.1 |  | Rsa10027428;Rsa10028034;Rsa10002250;Rsa10002251;Rsa10009433;Rsa10015945;Rsa10015930;Rsa10006493 | Bra039825;Bra039824;Bra039823;Bra004012;Bra016676;Bra039705;Bra023838 |  |
|  | TGG2 | AT5G25980.2 | 489446;917734 |  |  |  |
|  | TGG4 | AT1G47600.1 | 474221;882771 | Rsa10014059;Rsa10027577;Rsa10027551 | Bra030473;Bra014287 | Bol044760 |
|  | TGG5 | AT1G51470.1 | 474259;891388 |  |  | Bol031599 |
|  | ESP | AT1G54040.2 | 319834;337565 | Rsa10012280 | Bra037958;Bra039702 | Bol024137;Bol006380;Bol006378;Bol039072;Bol013374 |
|  | NSP1 | AT3G16400.1 | 897952;479094 |  | Bra040923 |  |
|  | NSP2 | AT2G33070.3 | 497400;482246 |  |  |  |
|  | NSP3 | AT3G16390.1 |  |  |  |  |
|  | NSP4 | AT3G16410.1 |  |  |  |  |
|  | NSP5 | AT5G48180.1 | 494872 | Rsa10027284 | Bra037506 |  |
|  | PEN2 | AT2G44490.1 | 903873;870958;486477 | Rsa10039354;Rsa10024072 | Bra004833 | Bol045598 |
|  | PEN3 | AT1G59870.1 | 475320;471713;318409;471753;482613;894406 | Rsa10029736;Rsa10040977;Rsa10037983;Rsa10032108;Rsa10038015;Rsa10020447 | Bra003527;Bra016669;Bra026156;Bra026157;Bra021173;Bra026124;Bra023065 | Bol021414;Bol029238 |
|  | CAD1 | AT5G44070.1 | 917217;333720 | Rsa10021597;Rsa10012304;Rsa10002898 | Bra033718;Bra027525;Bra036010;Bra027526;Bra032535 | Bol012856;Bol024165;Bol009509;Bol009508;Bol018370 |

**Table S2.** The syntenic relationships between AtGSLs genes and RsGSL genes.

| AT1G07640 | Rsa10019875;Rsa10003296;Rsa10041327 |
| --- | --- |
| AT1G18570 | Rsa10013845;Rsa10038229;Rsa10040860 |
| AT1G62540 | Rsa10033546 |
| AT1G65880 | Rsa10015752;Rsa10033891 |
| AT1G74080 | Rsa10030571;Rsa10034277 |
| AT1G74090 | Rsa10030573;Rsa10030574;Rsa10023491;Rsa10023488;Rsa10023484;Rsa10034274 |
| AT2G20610 | Rsa10031178 |
| AT2G31790 | Rsa10038489;Rsa10016624 |
| AT3G03190 | Rsa10003824 |
| AT3G09710 | Rsa10034552;Rsa10042918 |
| AT3G19710 | Rsa10031835 |
| AT3G49680 | Rsa10032850;Rsa10006817 |
| AT4G12030 | Rsa10009388 |
| AT4G23100 | Rsa10014287;Rsa10036547 |
| AT4G30530 | Rsa10019051;Rsa10010316 |
| AT4G39940 | Rsa10026307;Rsa10037346;Rsa10027011 |
| AT4G39950 | Rsa10027010 |
| AT5G05260 | Rsa10002991;Rsa10020966 |
| AT5G07690 | Rsa10036406;Rsa10020837 |
| AT5G14200 | Rsa10025187 |
| AT5G44070 | Rsa10012304;Rsa10021597 |
| AT5G57220 | Rsa10019377;Rsa10023813 |
| AT5G60890 | Rsa10019673;Rsa10018401;Rsa10033651 |
| AT5G61420 | Rsa10019636;Rsa10017936;Rsa10033628 |
| AT5G65940 | Rsa10016044 |

**Table S3.** The tandem repeat arrays of GSL genes in *R. sativus*.

| 1 | GGP1 | Rsa10023138 | Rsa10023137 |  |  |
| --- | --- | --- | --- | --- | --- |
| 2 | MYB34 | Rsa10019673 | Rsa10019672 |  |  |
| 3 | TGG1 | Rsa10002250 | Rsa10002251 |  |  |
| 5 | CYP81F2 | Rsa10026366 | Rsa10026367 |  |  |
| 6 | SUR1 | Rsa10022084 | Rsa10022083 |  |  |
| 7 | MYB122 | Rsa10030571 | Rsa10030572 |  |  |
| 8 | AOP1 | Rsa10022483 | Rsa10022481 |  |  |
| 10 | GSH1_PAD2 | Rsa10036547 | Rsa10036546 |  |  |
| 11 | GSL-OH | Rsa10016552 | Rsa10016553 |  |  |
| 14 | ST5b | Rsa10023491 | Rsa10023490 | Rsa10023489 | Rsa10023487 |
| 18 | ST5b | Rsa10022357 | Rsa10022358 |  |  |
| 16 | GGP1 | Rsa10010316 | Rsa10010315 |  |  |
| 17 | CYP81F4 | Rsa10026964 | Rsa10026966 |  |  |

1. Institute of Vegetables and Flowers, Chinese Academy of Agricultural Sciences; Key Laboratory of Biology and Genetic Improvement of Horticultural Crops, Ministry of Agriculture, Beijing 100081, China. ^2^ Beijing Genomics Institute, Shenzhen, Guangdong 518083, China. ^3^ Vegetable Research Center of the Beijing Academy of Agriculture and Forestry Sciences, Beijing 100097, China. *These authors contributed equally to this work. Correspondence and requests for materials should be addressed to L.X. (email: lixixiang@caas.cn). [↑](#footnote-ref-2)
